# Supplementary figures and images for: Biochemical and molecular characterization of N66 from the shell of Pinctada mazatlanica
Source: PeerJ. 2019 Jun 27;7:e7212. doi: 10.7717/peerj.7212 (PMC6599672; doi:10.7717/peerj.7212)

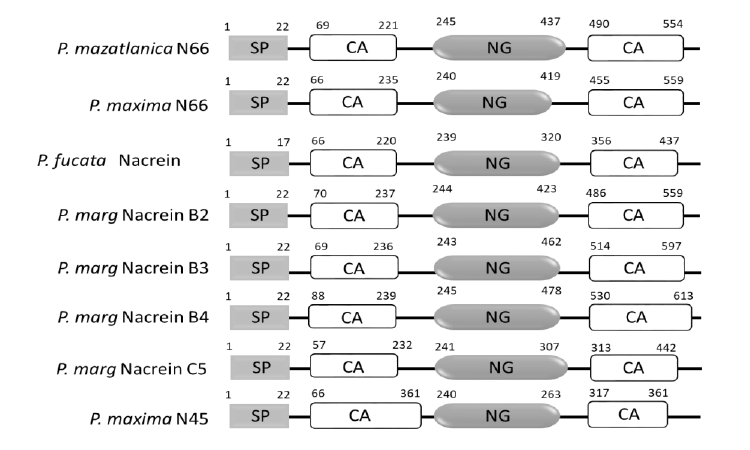

Supplement: Supplemental Information 1 — Signal peptide (SP), carbonic anhydrase domain (CA) and Asparagine-Glycine domain (NG domain) are shown. P. maxima N66 (GenBank: BAA90540.1), P. fucata nacrein (GenBank: BAA119401.1), P. margaritifera nacrein B2 (GenBank: ADY69618.1), B3 (GenBank: AEC03971.1), B4 (GenBank: AEC03972.1) and C5 (GenBank: AEC03973.1), and P. maxima N45 (GenBank: ACT55367.1). [file peerj-07-7212-s001.jpg]

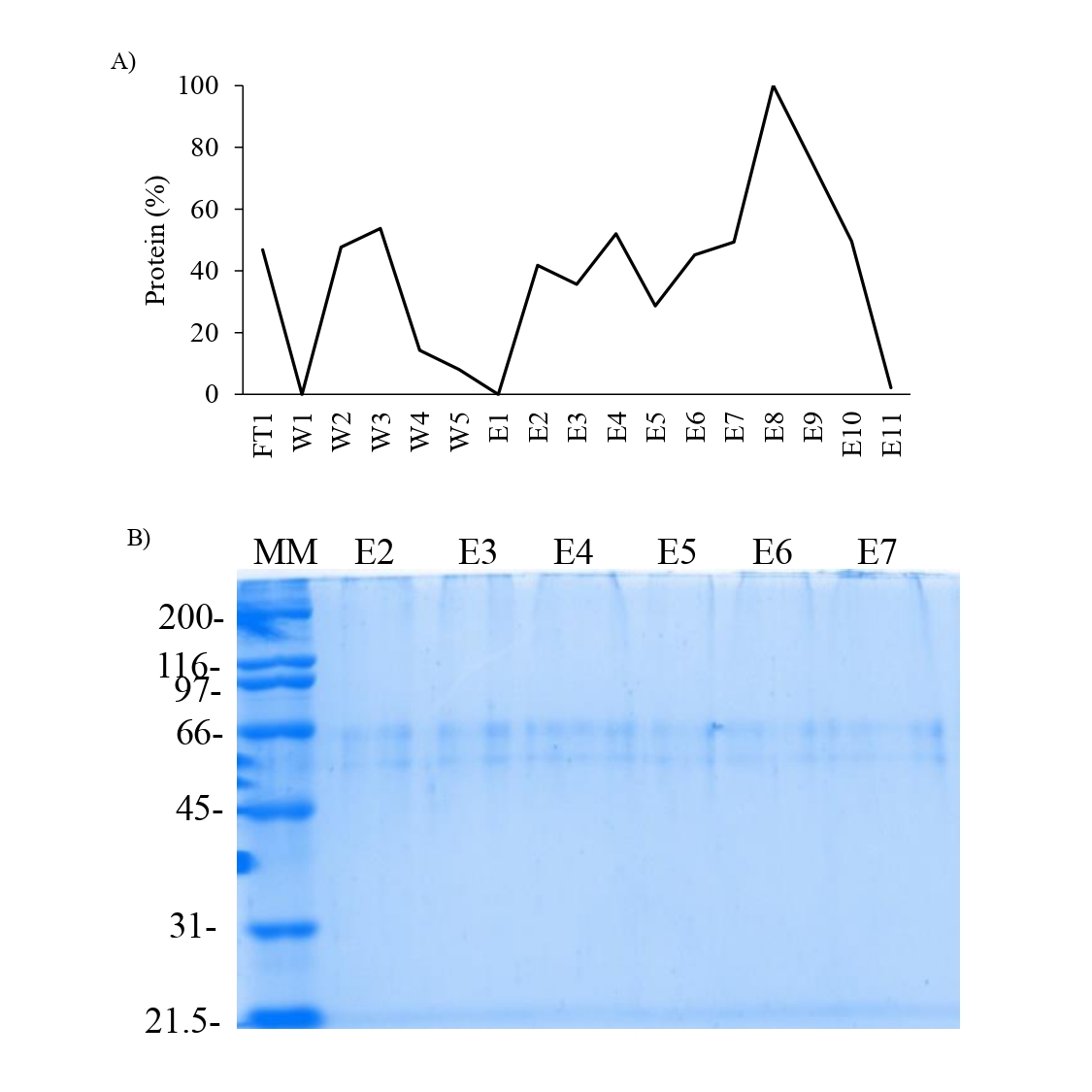

Supplement: Supplemental Information 2 — A) Chromatogram from the Sepharose-Ni column, B) SDS-PAGE 10% of the elution fraction derived of the affinity chromatography. MM: Molecular marker; FT: Flowthrough; W: wash; E: Elution fraction. [file peerj-07-7212-s002.jpg]

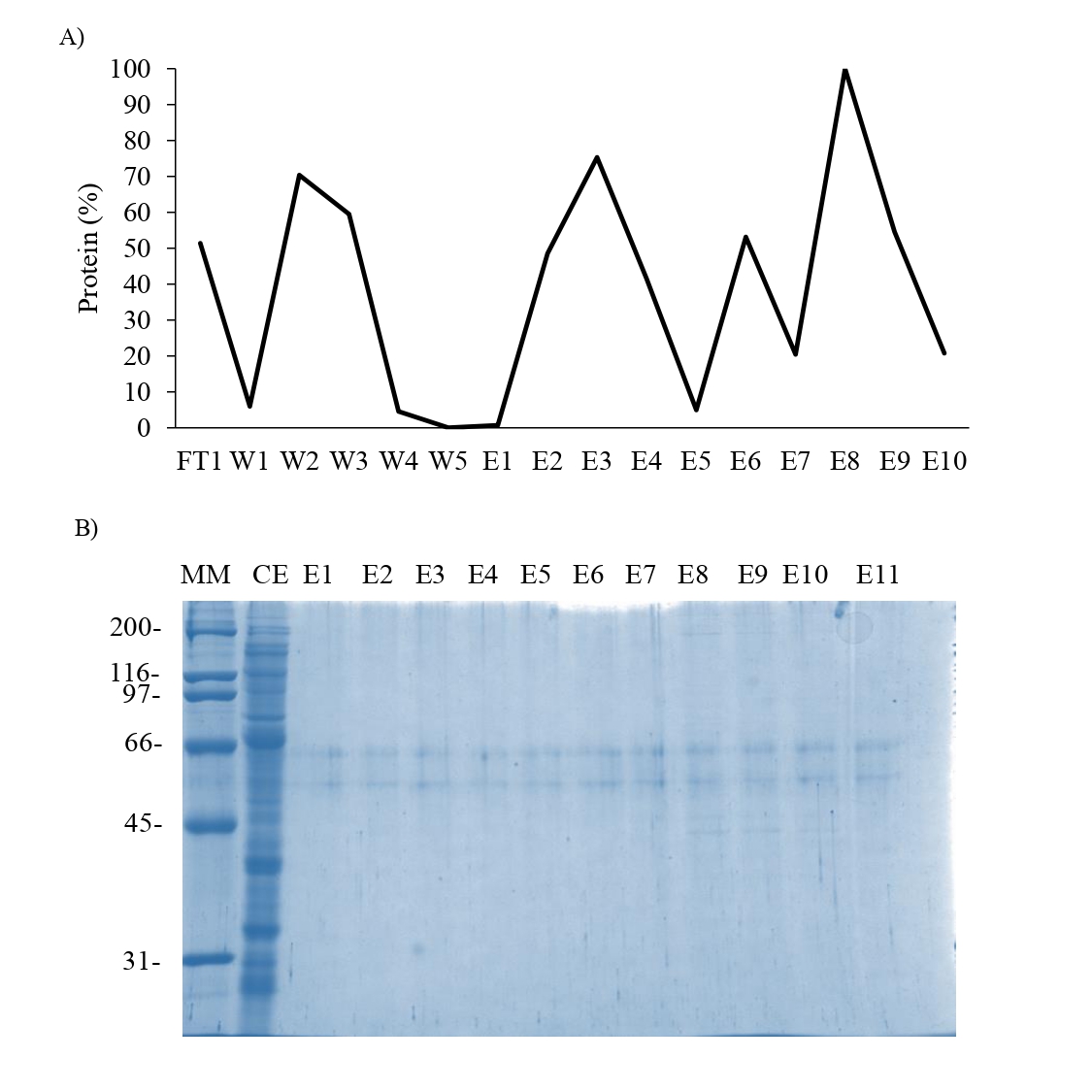

Supplement: Supplemental Information 3 — A) Chromatogram from the Sepharose-Ni column, B) SDS-PAGE 10% of the elution fraction derived of the affinity chromatography. MM: Molecular marker; CE: Crude extract; FT: Flowthrough; W: wash; E: Elution fraction. [file peerj-07-7212-s003.jpg]
