# Supplementary material for: Biochemical and molecular characterization of N66 from the shell of Pinctada mazatlanica
Source: PeerJ. 2019 Jun 27;7:e7212. doi: 10.7717/peerj.7212 (PMC6599672; doi:10.7717/peerj.7212)
Supplement: Supplemental Information 4 [file peerj-07-7212-s004.pdf]

N66 nucleotide sequence

TATGTGGAGAATGACGACGCTTCTTCACTTGACTGCTCTGCTTGTTCTGATTCCATTATGTCATTGTGCCT
CCATGCACAGGCATGACCATTATATGGACATGGATCAAACCTACCGTGATCGATGGGGAAACTGTCATTATTCAGG
GGGAAGTAGCTGTGACGCCGGGTTTAGCTACAATAGGGAACAAAATGAGGAACAATGCCACGGGCCGTATGACT
GGCACACTATATCTAGTTGCTTTAAGGCATGTGGAAGTAAAGAGAGACAATCACCAATCAACATTTGGTCACATAG
AGCCCTTTTCCGAAAACTGCCAAGACTGAAATTCAAGCCACATATGAAATCATTGGATACGAAAGTGTCAAATCAC
CAAAATCATGCCCCTGAATTCGATTCTGAGGACGAAAAACTTCATGTTAAACTGAAGAAACTTGTTGATGGACATT
ATAAATTCCGCAATCTCCATATTCACATTGGCAAAAGTAGACGAAAGGGCTCCGAACACAGCGTTGACAGACATTT
TACACCTATGGAGGCTCATTTAGTGTTCCATCATGATGAGAAAAAGGAAATCAAACCTCCTAGGATTCCGTTAGGA
AGAAATTTCAGTGGTATTAATGAATTTGTTGTCGTTGGGGTTTTTCTAGAGGATGGTGATGAAGGATACGGTGATG
AACCGGACGACTATGAATGTAAGCGCATATTAAAGGGTCATTACGATCATTGCGACAACAATGGAGACAACG
GTTACAACTGTGATAACGGCAACAATGAAAACAACGGAAACAATGGTAATGGTAACAACGGCTATAACGGT
AACAACGGTTATAACGGTAATAACGGTGACAATGGCAACAGTGGAAACAATGGTAATGGTAACAACGGTTA
TAACGGTGACAATGGCAACAGCGGAAACAATGGTAATGGTAACAACGGTAATAACGGTAATAACGGTGGC
AACGGAAACAACAGAAACAATGGTAATGGTAACAACGGTTATAACGGTAATAACGGTGATAATGGCAACAA
CGGAAACAATGGTAATGGTAACAACGGAAATAATGGTAATGGTAACAACGGAAATAACGGTGGCAATGGC
AACAACGGAAACAATGGTAATGGTAACAACGGAAATAATGGTAATGGTAACAACGGGAATAACGGTGGCA
ATGGCAACAATGGTAATGGTAACAATGGAAGTAATGGTAACGGTGACTACGGTAGTAATGGTAACAATGGT
GGAAACGGGAACAATGGTAATAACGGTGATAACGGTAATGGCGACAATGGTTATAACGGTGATAATGGTA
ACAGTGACGGGCGACTCAGACGTTGGGACTTGGAAAATGTCCGCCGCATTCATACCGAGCGATATCACAT
CAGCGGAAGATGTATTGTCAAAAAAGCAAAACGCCTCAGCAGGATTCTCGAATGCGCATATAGACACAAAA
AAGTCAGAGAATTCAAAAGGAATGGAGAACACAAAAGTCTTGATGTTGAAATTACACCGGAAATGGTTCTA
CCGCCAATAAAATACAGACAATACTATACCTATGAAGGATCTTTGACAACCCCTCCTTGCACAGAGAGCGT
CCGTTGGGTTGTAGAAAAATGCCACGTGCAAGTATCCAGAAGGGTGCTTGATGCATTGCGGAAGGTCGAA
GGATATGATGATGGTACCACGTTTGAGCAAGTATGGAAACAAGACGTCCCACACAGAGAAACATAAAACCT
GTACCTGTGTACAAAAACTTTATATGAGA

N66 Protein sequence (576 aa)

MWRM**TT**LLHLTALLVLIPLCHCASMHRHDHYMDMDQTYRDRWGNCHYSGGSSCDAGFSYNREQNEEQCHGPYD
WHTISSCFKACGSKERQSPINIWSHRALFRKLPRLKFKPHMKSLD**T**KVSNHQNHAPEFDSEDEKLHVKLKKLVDGHYKF
RNLHIHIGKSRRKGSEHSVDRHFTPMEAHLVFHHDEKKEIKPPRIPLGRNFSGINEFVVVGVFLEDGDEGYGDEPDDYEC
KRILKGHYDHCDNNGDNGYNCDNGNNENNGNNGNGNNGYNGNNGYNGNNGDNGNSGNNGNGNNGYNGDNG
NSGNNGNGNNGNNGNNGGNGNNRNNGNGNNGYNGNNGDNGNNGNNGNGNNGNNGNGNNGNNGGNGNN
GNNGNGNNGNNGNGNNGNNGGNGNNGNGNNGSNGNGDYGSNGNNGGNGNNGNNGDNGNGDNGYNGDN
GNSDGRLRRWDLENVRRIHTERYHISGRCIVKKAKRLSRILECAYRHKKVREFKRNGEHKSLDVEITPEMVLPPIKYRQYY
TYEGSLTTPPCTESVRWVVEKCHVQVSRRVLDALRKVEGYDDGTTFEQVWKQDVPHRET
